# Production of Lactate by Metabolically Engineered *Scheffersomyces stipitis*

**DOI:** 10.3390/jof11060413

**Published:** 2025-05-27

**Authors:** Angela Matanović, Nenad Marđetko, Ana Slišković, Blanka Didak, Karla Hanousek Čiča, Bojan Žunar, Anamarija Štafa, Božidar Šantek, Marina Svetec Miklenić, Ivan-Krešimir Svetec

**Affiliations:** 1 Laboratory for Biology and Microbial Genetics, Department of Biochemical Engineering, Faculty of Food Technology and Biotechnology, University of Zagreb, Pierottijeva 6, 10000 Zagreb, Croatia; angela.matanovic@gmail.com (A.M.); asliskovic@pbf.hr (A.S.); bojan.zunar@pbf.unizg.hr (B.Ž.); anamarija.stafa@gmail.com (A.Š.); 2 Laboratory for Biochemical Engineering, Industrial Microbiology and Malting and Brewing Technology, Department of Biochemical Engineering, Faculty of Food Technology and Biotechnology, University of Zagreb, Pierottijeva 6, 10000 Zagreb, Croatia; 3 Laboratory for Fermentation and Yeast Technology, Department of Food Engineering, Faculty of Food Technology and Biotechnology, University of Zagreb, Pierottijeva 6, 10000 Zagreb, Croatia; karla.hanousek.cica@pbf.unizg.hr

**Keywords:** microbial lactate production, *Scheffersomyces stipitis*, non-conventional yeast, L-lactate dehydrogenase, metabolic engineering, xylose fermentation, codon-optimized gene expression, yeast cell factory

## Abstract

Lactate is a valuable compound used in food, chemical, and pharmaceutical industries. High-value, optically pure L- or D-lactate can be synthesized microbially via specific dehydrogenases. The non-conventional yeast *Scheffersomyces stipitis*, which is known for fermenting both hexoses and pentoses, is a promising host for biochemical production from lignocellulosic biomass but does not naturally produce lactate. In this study, we engineered *S. stipitis* to produce lactate by expressing two codon-optimized bacterial L-lactate dehydrogenase genes under the control of strong native promoters. The engineered strain produced 7.42 g/L (0.46 g/g yield) and 11.67 g/L (0.58 g/g yield) lactate from glucose and xylose, respectively. The highest titer, 19.27 g/L (0.52 g/g yield), was achieved from 50 g/L xylose after 74 h. Increasing the fermentation temperature from 28 °C to 32 °C improved yield by 30%, while a neutralizing agent further enhanced yield by 25% and prevented lactate degradation following carbon depletion. Although the wildtype strain produced a significant amount of ethanol on both glucose and xylose, the engineered strain produced ethanol as a side product exclusively on glucose and not on xylose. This phenomenon could be advantageous for biotechnological applications and may reflect shifts in gene expression depending on the carbon source or even on the presence of lactate.

## 1. Introduction

Given the state of plastics pollution on land and in the oceans, it is becoming imperative that in instances where plastic cannot be avoided, biodegradable plastic should be used. Bioplastics such as polylactic acid (PLA) are not based on the fossil fuel industry, since they can be produced from biotechnologically derived monomers. For the production of PLA, the basic monomer that is used is L-lactic acid. L-lactic acid is also commonly used as a pH modulator in food and feeds, in pharmaceutical and cosmetics industries, and as a chiral precursor for production of various chiral compounds [1,2,3].

The advantage of microbial synthesis of lactic acid over chemical synthesis is that it is much simpler to achieve a product that is highly pure, both optically and chemically (which is far more valuable than racemic lactic acid and has a broader range of applications), via microbial synthesis. Currently, optically pure lactic acid is mostly produced by bacterial fermentation, using either lactic acid bacteria (LAB) or metabolically engineered strains of *Escherichia coli* [4,5,6,7,8]. Some homofermentative bacteria LAB ferment sugars into lactate with almost theoretical yield (nearly 1 g/g) and high titers (more than 100 g/L). The main drawback of bacterial fermentation is its poor tolerance to a low pH medium and the cost of the medium [6]. Currently, most lactic acid production is based on submerged fermentation of corn [9], but other raw materials, such as various byproducts of the food industry and lignocellulose biomass, are being intensely investigated [3]. Some of these issues can be bypassed using filamentous fungi and yeasts as working organisms. Fungi from the *Rhizopus* genus (*R. oryzae* and *R. arrhizus*) can produce lactic acid and have several advantages over LAB: they demonstrate amylolytic activity, so the saccharification step of starchy substrate can be omitted; they can grow on simple media; and they grow as pellets or filaments, which simplifies biomass separation. Titers and yields vary depending on the strain and the process parameters, but they can be up to 173 g/L and 0.94 g/g on glucose [5]. At the same time, filamentous or floccular growth negatively affects aeration and, thus, the productivity of the process [5,10].

Although yeasts cannot produce lactic acid naturally, they can be engineered to be lactate producers. Additionally, compared to bacteria, yeasts are much more tolerant of a lower pH, which reduces the need for neutralizing agents and reduces downstream processing costs [5]. Lactate dehydrogenases from various sources have been expressed in *Saccharomyces cerevisiae* [5,11,12], *Candida* sp. [13,14,15], *Kluyveromyces* sp. [16,17], *Pichia stipitis* [18], *Yarrowia lipolytica* [19], and *Zygoaccharomyces bailii* [20]. In some of these yeasts, additional modifications were made to increase lactate yield, with varying success. Some of the best yeast producers are engineered *Kluyveromyces lactis*, achieving 109 g/L and 0.91 g/g, and *Candida sonorensis*, achieving 92 g/L and 0.94 g/g, both on glucose [5].

*Scheffersomyces* (*Pichia*) *stipitis* is a non-conventional yeast species which was isolated from rotting wood and from insects living and feeding on such material [21]. It attracted researchers’ attention due to its natural ability to efficiently ferment both pentoses and hexoses and produce ethanol. Most notably, unlike the commonly used workhorse in biotechnology yeast, *Saccharomyces cerevisiae*, *S. stipitis* can efficiently ferment D-xylose, a major pentose in hemicellulosic plant material. Efficient fermentation of D-xylose is a feature characteristic to a few yeast species belonging to the *Scheffersomyces* clade, *Spathaspora* clade, and *Pachsolen* tannophulus [21]. Therefore, this yeast species can be used for sustainable production of biochemicals and bioethanol from lignocellulose material containing significant amounts of D-xylose [22]. *S. stipitis*, like the majority of other yeast species, does not produce lactate naturally. *S. stipitis* was successfully engineered to produce L-lactate by expressing L-lactate dehydrogenase from *Lactobacillus helveticus* under the control of the *ADH1* promoter and the *CYC1* terminator from *S. cerevisiae* [18]. In addition, in other yeasts, it has been shown that co-expression of two different L-lactate dehydrogenases results in a higher yield of L-lactate [16]. The explanation for this result could be that the two enzymes demonstrate optimum activity in different conditions during the progression of fermentation, such as different pH optima, and together, they achieve a higher product yield.

The goal of our study was to construct a strain of *S. stipitis* that is able to efficiently produce L-lactate. In our previous study, we identified two L-lactate dehydrogenases (*Idh1* and *Idh2*) in the genome of *Lactobacillus gasseri* and experimentally confirmed their L-lactate dehydrogenase function [23]. To utilize the advantages of *S. stipitis* yeast for lactate production, in this study, *IdhL1* and *IdhL2* genes from *Lactobacillus gasseri* were codon-optimized and co-expressed under the control of strong native *S. stpitis PDC1* and *TDH3* constitutive promoter–terminator pairs [24]. Lactate production was tested on glucose, xylose, or both sugars (as two main carbon sources present in renewable substrates), with variation in several fermentation parameters. The constructed strain efficiently produced lactate when growing on either or both of these sugars, achieving generally higher yields than those produced by previously available strains (Ilmen et al., 2007 [18]) under similar fermentation conditions. Furthermore, the addition of neutralizing agent, as well as increasing the fermentation temperature from 28 °C to 32 °C, positively affected the lactate yield.

## 2. Materials and Methods

### 2.1. Microorganisms

Bacteria *Escherichia coli* strain DH5α was used for plasmid construction, maintenance, and isolation [25]. The starting strain for the construction of the L-lactate producer was *Scheffersomyces stipitis* JCM 10742^T^, a type of strain obtained from the Japanese Collection of Microorganisms.

### 2.2. Media and Growth Conditions

*E. coli* was grown at 37 °C and 150 rpm in LB-Miller medium (10 g/L bacto tryptone, 5 g/L yeast extract, 10 g/L NaCl) and 15 g/L of agar for solid media. For the selection and maintenance of transformants, the medium was supplemented with ampicillin (50 µg/mL in liquid medium, 100 µg/mL in solid medium, Fisher BioReagents, Pittsburgh, PA, USA).

*S. stipitis* was grown at 28 °C and 150 rpm in YPD (10 g/L yeast extract, 20 g/L bacto peptone, 20 g/L glucose) with the addition of 20 g/L of agar in solid medium. For the selection and maintenance of transformants, the medium was supplemented with 400 µg/mL of hygromycine B (Fisher BioReagents, Pittsburgh, PA, USA). Additionally, in some experiments, YPD was supplemented with 14 g/L of CaCO_3_. When xylose was used as a main carbon source during L-lactate production, the glucose in YPD was replaced with either 20 g/L or 50 g/L of xylose (this medium is named YPX20 or YPX50, respectively). For co-fermentation, a medium with glucose and xylose, both at 25 g/L, was used. Media for yeast and bacteria were prepared as described by Sambrook and Russel [26].

For lactate production, *S. stipitis* wildtype (control) or engineered Lac^+^ producer preculture was grown in 2 mL of YPD or YPX20 until the stationary phase growth was reached (48–72 h). Next, 1 mL of preculture was inoculated in 100 mL of fresh medium prepared in a 500 mL Erlenmeyer flask. The first sample for UPLC analysis was collected immediately after inoculation. This sample was centrifuged at 5000 rpm for 15 min to precipitate most of the yeast cells, and the resulting supernatant was preserved by freezing for subsequent analysis. The initial number of colony-forming units was determined by preparation of decimal dilutions and inoculation on YPD plates. The culture was then incubated on an orbital shaker at 150 rpm and 28 °C, and samples were obtained at specific time points (as indicated on graphs on as indicated in the Section 3), centrifuged at 5000 rpm for 15 min, and frozen for later analysis. At each sampling point, the total number of living cells was determined by plating suitable decimal dilutions on YPD plates.

### 2.3. Plasmid Construction

**Figure 1:**
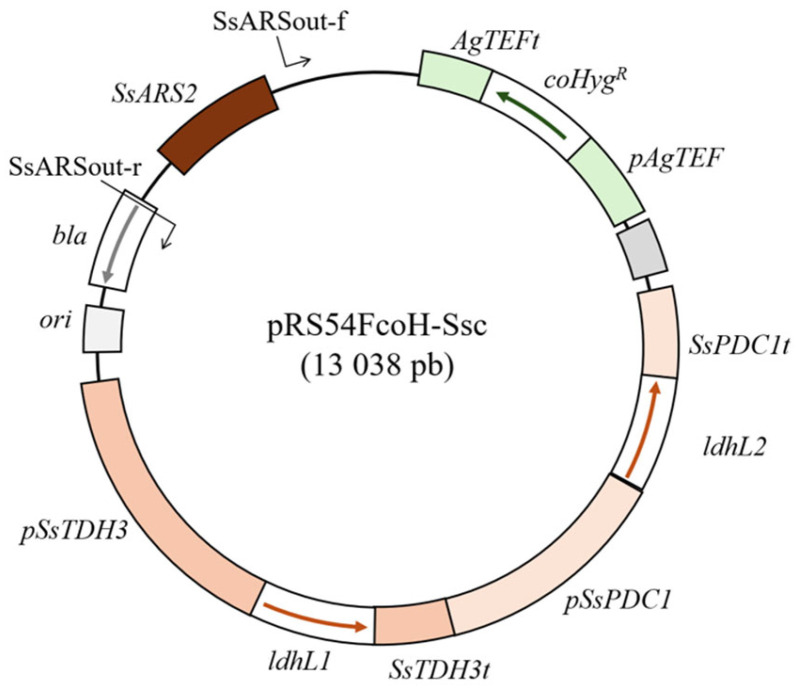
Map of pRS54FcoH-Ssc plasmid. Relevant plasmid regions and primer annealing sites are marked. Detailed explanations are provided in the text.

Genes *IdhL1* and *IdhL2* encoding L-lactate dehydrogenases originating from the bacteria *Lactobacillus gasseri JCM1131* were codon-optimized using an online tool from Integrated DNA Technologies (https://www.idtdna.com/pages/tools/codon-optimization-tool, accessed on 21 December 2022). The ORFs were codon-optimized using *Candida albicans* codon preference, since this yeast also belongs to the CTG clade and is relatively closely related to *S. stipitis*. The expression of *IdhL1* genes was put under the control of native *S. stipitis TDH3* (glyceraldehyde-3-phosphate dehydrogenase) and the *IdhL2* was put under the control of native *S. stipitis PDC1* (pyruvate decarboxylase) promoter–terminator pair. These promoter–terminator pairs were chosen on the basis of transcriptome analysis of *S. stipitis* growing on glucose or xylose, which demonstrated that both *PDC1* and *TDH3* are strongly and constitutively expressed [24]. This entire segment carrying two consecutive codon-optimized genes for L-lactate dehydrogenases (*TDH3* promoter–codon-optimized *IdhL1* ORF-*TDH3* terminator and *PDC1* promoter–codon-optimized *IdhL2* ORF-*PDC1* terminator) was synthesized by Genewiz (www.genewiz.com) and cloned into standard pUG vector. The exact sequences of synthesized fragments are provided in Supplementary Figure S1. The fragment was excised from this vector using PvuII endonuclease and cloned into the PvuII restriction site of the pRS54FcoH vector, thus creating pRS54FcoH-Ssc, which will be used for yeast transformation. The pRS54FcoH vector originates from the pRS54coH which we described in our previous work [27]. The pRS54coH carries origin of replication and codon-optimized hygromycin B yeast selection marker expressed under the *TEF* promoter. The difference between the pRS54coH described earlier and pRS54FcoH used in this work is that the *f1 ori* (enabling plasmid isolation in ssDNA form) was inactivated by removing part of this region from the vector backbone. A schematic representation of the pRS54FcoH-Ssc plasmid which was used for amplification of transforming DNA is shown in Figure 1. All buffers and standard molecular genetics techniques were performed as described by Sambrook, J. and Russell, D.W. [26].

### 2.4. Yeast Transformation, Stability of Transformants, and Molecular Analysis

Yeast was transformed with a linear fragment obtained by PCR using SsARS-out-f (ctttctccttctcgttagcatc) and SsARS-out-r (ttccgtgtcgcccttattc) primers and pRS54FcoH-Ssc as the template (the position of the primer annealing is shown in Figure 1). Thus, a linear integrative 11,668 bp fragment which has all the regions of the circular pRS54FcoH-Ssc except the origin of replication was amplified. Transformation of *S. stipitis* was carried out via electroporation [28], and transformants were selected on plates supplemented with hygromycin B (the total concentration of hygromycin B for transformant selection was 400 µg/mL). *S. stipitis* was transformed with efficiency of 5.2 × 10^2^ transformants/µg of DNA.

From three randomly selected transformants, total genomic DNA was isolated and digested with PshAI endonuclease. The integration of the transformation vector was verified by Southern blotting, where the DIG-labeled hygromycin B resistance gene served as the probe. The synthesis and labeling of the probe and the Southern blotting procedure were conducted according to methods previously described by Štafa et al. [29], utilizing the Roche DNA Labelling and Detection Kit (F. Hoffmann-La Roche Ltd., Basel, Switzerland).

To test whether transformants were stable, a single colony of the strain was grown in 100 mL of non-selective medium until the stationary phase. The resulting cell suspension was then plated onto non-selective plates to obtain approximately 100 colonies. These colonies were replica-plated onto solid selective medium YPD with 400 µg/mL of hygromycine B (Fisher BioReagents, Pittsburgh, PA, USA). The strain was considered stable if all of the replicated colonies were able to grow on selective media.

### 2.5. Sample Preparation and UPLC Analysis

When the samples were collected as described in Section 2.2, 750 µL of a 10% solution of ZnSO_4_ was added to 750 µL of each sample. The mixtures were incubated for 10 min at room temperature to allow protein precipitation and then centrifuged at 13,000 rpm for 5 min. The supernatant was subsequently filtered into vials through a nylon syringe filter (CHROMAFIL Xtra PA-20/25, Duren, Germany) with a pore diameter of 0.2 μm. The prepared samples were analyzed by liquid chromatography (Agilent Technologies 1290 Infinity II, Santa Clara, CA, USA). A volume of 10 μL of each sample was injected into the UPLC system. An analytical column, Rezex ROA-Organic Acid H^+^ (15 cm × 7.2 mm, Phenomenex, Torrance, CA, USA), was used for the analysis. Elution was performed isocratically with 0.0025 M H_2_SO_4_ as the mobile phase at a flow rate of 0.6 mL min⁻^1^. The column oven temperature was set to 60 °C, and the temperature of the RI detector was set to 40 °C.

### 2.6. Statistycal Analysis

All fermentations were performed at least two independent times, including the independent medium and inoculum preparation. Average values and standard deviations were calculated and are presented on the graphs.

## 3. Results

### 3.1. Construction and Selection of Lactate-Producing Strains

To construct the L-lactate-producing strain, two genes (*ldhL1* and *ldhL2*) encoding L-lactate dehydrogenases from the bacteria *Lactobacillus gasseri JCM1131* were codon-optimized and cloned (as described in Section 2.3 and Section 2.4). *L. gasseri* was chosen because we previously determined that it has an excellent capacity for L-lactate production [23].

To achieve strong constitutive expression of codon-optimized ORF-s of *ldhL1* and *ldhL2* genes, we used *S. stipitis* native promoters and terminators of genes belonging to the central carbon metabolism *(PDC1* and *TDH3)* which are strongly and constitutively expressed during growth on glucose or xylose in both aerobic and oxygen-limiting conditions, as shown by transcriptome analysis [24,30].

*S. stipitis* was transformed with linear integrative fragment and a subset of transformants was subjected to molecular analysis by Southern blotting (Supplementary Figure S2) which confirmed a random integration into the genome. Since the strains were constructed via illegitimate (non-homologous) recombination, we expected them to be highly stable (which was experimentally confirmed as described in Section 2.4). Moreover, because of the random integration site in the *S. stipitis* genome, we could anticipate that the level of gene expression might vary between different transformants. Differences in gene expression levels could arise if, for example, the transforming DNA was integrated into a part of the genome featuring more condensed chromatin, such as near centromeres or telomeres. For this reason, we initially tested three transformants to determine if and how much lactate each of them produces. The transformants, together with non-transformed *S. stipitis* as a control, were inoculated in YP medium prepared with 20 g/L of glucose, 20 g/L of xylose, or 50 g/L of xylose, and lactate production was measured at various time points during growth. The best producer in a preliminary experiment produced 7.24 g/L of lactate (while the other two produced 6.66 g/L and 6.98 g/L) on 20 g/L glucose. On 20 g/L xylose, the same transformant produced 11.58 g/L of lactate (the other two produced 7.96 g/L and 8.11 g/L), while on 50 g/L xylose, the best transformant produced 20.64 g/L (the other two produced 16.19 g/L and 17.34 g/L). Since this transformant (corresponding to Well 2 on the Southern blotting) consistently produced the highest concentration of lactate in all these fermentations, it was used in all further experiments described in this paper. We have named this lactate-producing strain *S. stipitis* Lac^+^.

### 3.2. Conversion of Glucose to Lactate by S. stipitis

**Figure 2:**
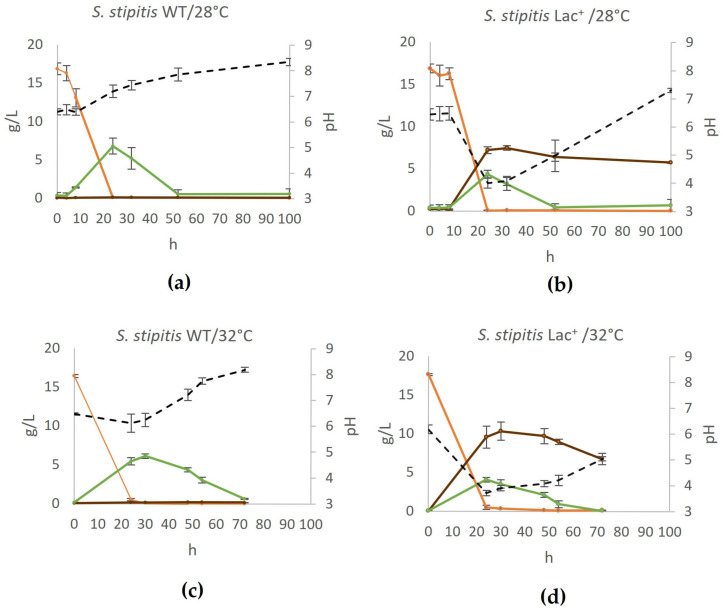
Conversion of glucose to lactate by *S. stipitis* in (**a**) wildtype at 28 °C, (**b**) engineered lactate producer at 28 °C, (**c**) wildtype at 32 °C, (**d**) engineered lactate producer at 32 °C. During fermentation on YPD, mass concentrations of glucose (orange), ethanol (green), and lactate (brown) were determined and are shown on the graphs, as well as the pH of the medium (black). Error bars represent standard deviation. Acetic acid and glycerol concentrations were also determined but were negligible at all time points.

To analyze the potential of the constructed strain to convert glucose to lactate, we used a standard YPD prepared with 20 g/L glucose and cultured the cells as described in Section 2.5. The experiments were conducted at temperatures of 28 °C and 32 °C to assess the impact of temperature on L-lactate production, and wildtype (WT) and engineered (Lac^+^) strains were compared (Figure 2).

As shown in Figure 2, the highest concentration of lactate produced from 20 g/L glucose (equals approx. 16 g/L after medium sterilization by autoclaving) was 7.42 g/L (yielding 0.46 g/g glucose), which was achieved after 32 h of fermentation. After that, the concentration was slightly reduced to 5.70 g/L lactate (after 100 h of fermentation). Most likely, after other substrates are exhausted, lactate is used as the carbon source. Increasing the temperature to 32 °C further enhanced L-lactate production by approximately 30% in the engineered Lac^+^ strain, reaching a peak concentration of 10.33 g/L at 30 h, corresponding to a yield of 0.64 g/g glucose. At the same time, the growth rate of the Lac^+^ strain was not compromised (Section 3.5). This underlines the need to consider the optimum temperature of heterologous enzymes originating from different species when testing the fermentation parameters. Initially, we also conducted tests at 37 °C, which is the optimal growth temperature for *L. gasseri* [31], but this resulted in slowed growth (both in the wildtype and Lac^+^ strain) and a lactate yield that was slightly less than at 28 °C.

The production of glycerol in *S. stipitis* used in this work is negligible, and the only other product originating from pyruvate, aside from lactic acid, is ethanol (Figure 2). The production of lactate reduces maximum ethanol concentration (from 6.8 g/L in WT to 3.99 g/L in Lac^+^ strain at 28 °C and from 6.11 g/L to 4.09 g/L at 32 °C). This illustrates that the cloned L-lactate dehydrogenases are very active and channel a significant amount of the pyruvate to lactate.

**Figure 3:**
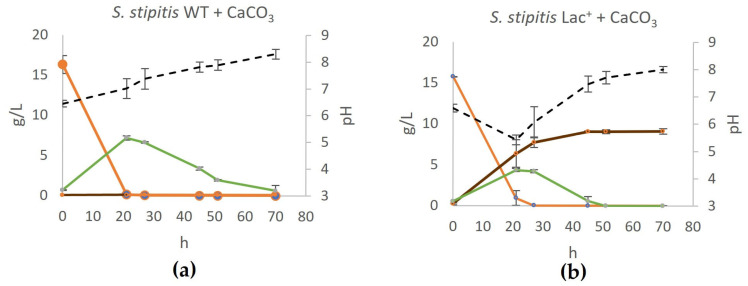
Production of lactate on CaCO_3_-buffered YPD in (**a**) wildtype and (**b**) engineered lactate producer. During fermentation on YPD supplemented with CaCO_3_ and during fermentation on YPD, mass concentrations of glucose (orange), ethanol (green), and lactate (brown) were determined and are shown on the graphs, as well as the pH of the medium (black). Error bars represent standard deviation.

Moreover, the production of lactate acidifies the medium, which could have a negative impact on cell growth and, thus, on lactate production. Lee et al. [16] showed that the production of lactate by genetically engineered *Kluyveromyces marxianus* increased 150% when CaCO_3_ was added to neutralize the medium during fermentation. To test if similar effects would be achieved, we added 14 g/L of CaCO_3_ into YPD and performed the fermentation at 28 °C (Figure 3a,b).

The presence of CaCO_3_ as a neutralizing agent had a positive impact on lactate production and the maximum yield increased in this case by 25% and was 9.17 g/L (0.57 g lactate/g glucose). Interestingly, in terms of medium neutralization, after the lactate accumulated to nearly maximum concentration (around 45 h of fermentation), the concentration remained constant, which is an additional advantage of using CaCO_3_.

### 3.3. Conversion of Xylose to Lactate by S. stipitis

**Figure 4:**
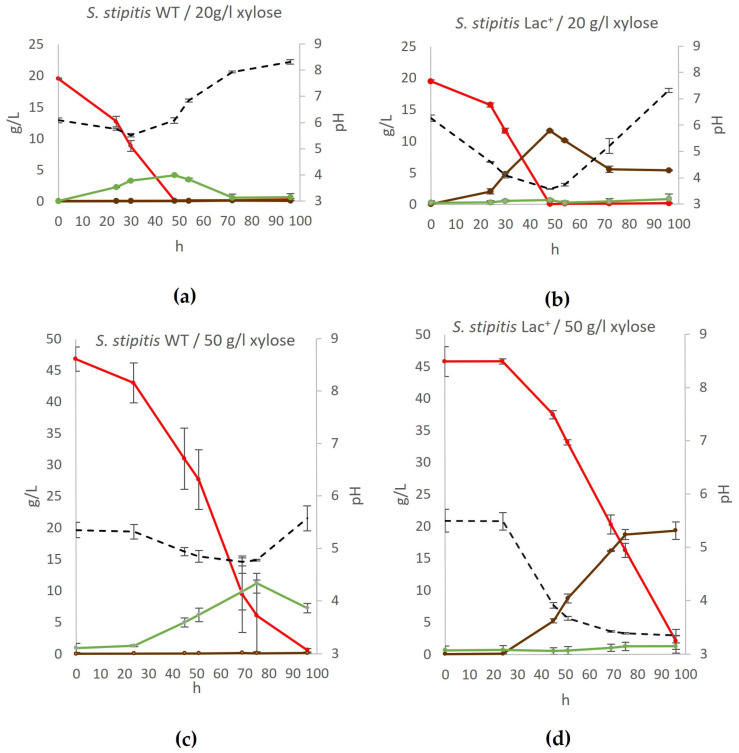
Conversion of xylose to lactate by *S. stipitis* for (**a**) wildtype yeast on YPX with 20 g/L xylose, (**b**) engineered lactate producer on YPX with 20 g/L xylose, (**c**) wildtype yeast on YPX with 50 g/L xylose, and (**d**) engineered lactate producer on YPX with 50 g/L xylose. During fermentation, mass concentrations of xylose (red), ethanol (green), and lactate (brown) were determined, as well as the pH of the medium (black). Error bars represent standard deviation. Acetic acid, glycerol, and xylitol concentrations were also determined but were negligible at all time points.

The ability of the engineered *S. cerevisiae* to produce lactate on YPX medium prepared with 20 g/L xylose or 50 g/L xylose was tested. The growth and fermentation processes in these media were monitored over 96 h, and the results are shown in Figure 4.

The results showed that the engineered strain converted xylose to lactate even more efficiently than glucose: 11.67 g/L of lactate was produced on YPX with 20 g/L xylose (0.58 g lactate/g xylose) and 19.27 g/L of lactate on YPX with 50 g/L xylose (0.52 g lactate/g xylose). As with glucose, when 20 g/L of xylose was added into the medium, the lactate concentration peaked (around 48 h of fermentation) and then gradually declined to 5.3 g/L after 96 h of fermentation.

It is intriguing that the Lac^+^ strain produces more L-lactate through xylose fermentation at 28 °C compared to glucose fermentation, achieving a yield of 0.58 g/g xylose in the YPX20 medium and 0.43 g/g in the YPX50 medium. When 50 g/L of xylose was added, the maximum lactate concentration was recorded at the last time point (96 h), since it took much longer for the substrate to be used up.

While the Lac^+^ strain successfully produces L-lactate, the yield is inversely related to the xylose concentration. At lower xylose concentration (20 g/L), the Lac^+^ strain achieves a higher yield of L-lactate, as the metabolic pathways may have a reduced load, allowing for more efficient conversion of substrate to product. However, at higher xylose concentration (50 g/L), despite an increase in absolute L-lactate production, the yield per gram of substrate (xylose) consumed is lower.

In fermentations performed on glucose (YPD medium), the main byproduct was ethanol. The wildtype produced up to 7 g/L of ethanol, while the engineered lactate-producing strain yielded around 4 g/L of ethanol (regardless of CaCO_3_), which illustrates the competitiveness of lactate and ethanol production in vivo. However, when fermentation was performed on xylose (YPX medium), ethanol production in the engineered strain was negligible, while the wildtype produced 4.2 g/L or 11.2 g/L ethanol (on YPX with 20 g/L or 50 g/L xylose, respectively). Moreover, this effect cannot be explained by conversion of ethanol to acetic acid, since no significant concentrations of acetic acid were recorded. Ilmén et al. [18] also observed a similar phenomenon during xylose fermentation using the strain expressing L-lactate dehydrogenase from *L. helveticus*. The authors hypothesized that higher concentrations of ethanol are not detected in the fermented medium because the ethanol produced is practically simultaneously consumed along with xylose.

**Figure 5:**
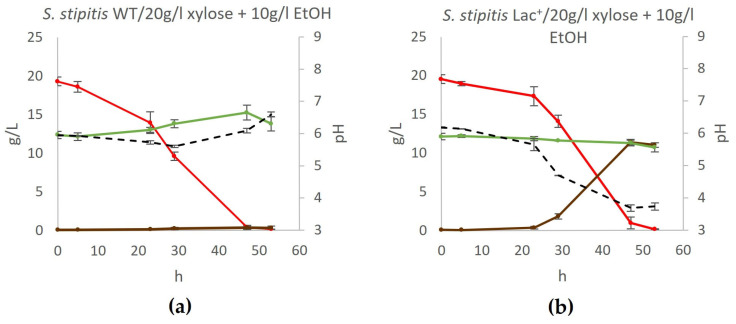
Fermentation of xylose medium supplemented with ethanol. During fermentation, mass concentrations of xylose (red), ethanol (green), and lactate (brown) were determined, as well as the pH of the medium (black) in (**a**) wildtype yeast and (**b**) engineered lactate producer. Acetic acid, glycerol, and xylitol concentrations were also determined but were negligible at all time points. Error bars represent standard deviation.

Therefore, we investigated whether the L-lactate producer ceases ethanol production or if ethanol is indeed generated but promptly metabolized despite the presence of xylose in the medium. This was investigated by cultivating the Lac^+^ strain in the YPX20 medium supplemented with 10 g/L ethanol (YPX20 + EtOH medium), and the obtained results are depicted in Figure 5.

As depicted in Figure 5, neither the wildtype yeast nor the Lac^+^ strain consumed ethanol if xylose was present in the medium. This leads to the conclusion that the Lac^+^ strain, in fact, does not produce ethanol during xylose fermentation.

### 3.4. Co-Fermentation of Glucose and Xylose by S. stipitis

**Figure 6:**
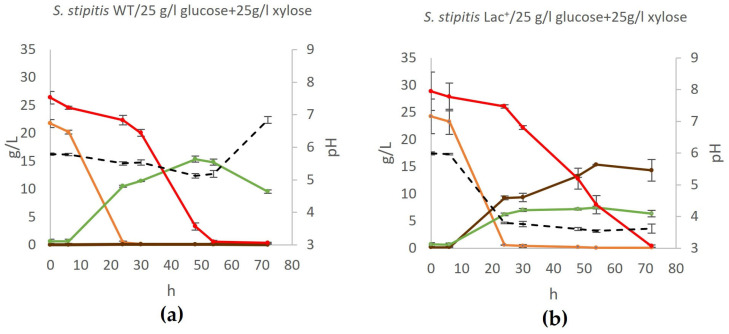
Production of lactate by co-fermentation of glucose and xylose. During fermentation, mass concentrations of glucose (orange), xylose (red), ethanol (green), and lactate (brown) were determined, as well as the pH of the medium (black) in (**a**) wildtype yeast and (**b**) engineered lactate producer. Acetic acid, glycerol, and xylitol concentrations were also determined but were negligible at all time points. Error bars represent standard deviation.

Glucose and xylose are the most abundant sugars derived from plant biomass, making their simultaneous utilization a key factor in enhancing the efficiency and economic viability of biotechnological processes for biochemical production. The fermentation medium, YPDX, contained equal concentrations of both sugars, summing up to a total sugar concentration of 50 g/L (25 g/L of glucose and 25 g/L of xylose), prior to sterilization. The cultivation of both the Lac^+^ strain and the wildtype strain in YPDX medium lasted for 72 h (Figure 6).

Although the yeast *S. stipitis* is capable of co-fermenting glucose and xylose, as confirmed in this study, the Lac^+^ strain achieves significantly lower yields in media containing both monosaccharides compared to media containing only one of these sugars. While co-fermentation does occur in the first 24 h of cultivation, glucose is still predominately used, while xylose concentration reduces by about 20%. During this phase of growth, both lactate and ethanol are produced. After the exhaustion of glucose, xylose is used, and lactate concentration further increases. However, consistent with experiments described above, during growth on xylose as the main carbon source, no further ethanol is produced, nor is ethanol used as a carbon source, despite the continued aeration of the flask.

### 3.5. Impact of Genetic Modifications on Growth Dynamics of S. stipitis Lac^+^ Strain

**Figure 7:**
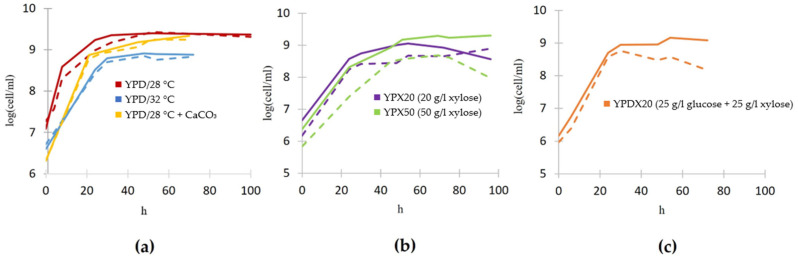
Growth curves of wildtype *S. stipitis* (full line) and genetically engineered Lac^+^ strain (dashed line), designed for L-lactate production, in different growing conditions. (**a**) YPD at 28 °C (red), YPD at 32 °C (blue), YPD supplemented with CaCO_3_ (yellow), (**b**) YPX20 (purple), YPX50 (green), and (**c**) YPDX (orange). Standard deviations are not shown for clarity of the graph and to avoid multiple overlapping error bars, but they were calculated and were up to +/−5% of the mean value.

Growth analysis indicates that the genetic modifications introduced to the Lac^+^ strain to enable L-lactate production do not adversely affect its growth rate on glucose compared to the wildtype (Figure 7a). Furthermore, the wildtype maintains a relatively stable growth curve across the different xylose concentrations, suggesting an inherent robustness to xylose utilization without the metabolic burden of enhanced L-lactate production (Figure 7b). When growing on 50 g/L xylose, the genetically modified Lac^+^ strain demonstrates some differences in growth patterns compared to the wildtype (Figure 7b). During growth in a medium that contains both glucose and xylose, the wildtype and Lac^+^ strain initially consume glucose (Figure 6), leading to uniform growth rates between them (Figure 7c). Around the 25th hour, after glucose is exhausted and they switch to consuming xylose, their growth curves somewhat deviate one from another, which could be indicative of metabolic stress due to the dual burden of xylose assimilation and L-lactate production.

## 4. Discussion

In this study, we expressed two codon-optimized L-lactate dehydrogenases originating from *Lactobacillus gasseri* in the yeast *Scheffersomyces stipitis*, which can naturally ferment both hexoses and pentoses. We used laboratory-scale flask fermentation to assess the abilities of the constructed strain to produce lactate on glucose, xylose, or both in comparison to wildtype *S. stipitis*. Other side products of the central carbon metabolism were also measured (acetate, glycerol, xylitol, and ethanol), of which only ethanol was detected. Wildtype *S. stipitis* did not produce any lactate, while the constructed strain produced 7.42 g/L (0.46 g/g yield) from glucose and 11.67 g/L (0.58 g/g yield) lactate from xylose, respectively. The highest titer, 19.27 g/L (0.52 g/g yield), was achieved from 50 g/L xylose.

When comparing the data on lactate production by Lac^+^ *S. stipitis* obtained in this study with the data from the literature on lactate production of the only previously known lactate-producing *S. stipitis* (constructed by Ilmen et al. [18] by expressing L-lactate dehydrogenase from *Lactobacillus helveticus* under a promoter and terminator originating from *Saccharomyces cerevisiae*), in terms of a comparison between the performance of the two strains in the most similar fermentation conditions, we can conclude that the strain described in this study produces lactate more efficiently: it produces 0.46 g/g of lactate while fermenting glucose, compared to 0.14 g/g by the strain of Ilmen et al. When CaCO_3_ was added as a pH-neutralizing agent, the yield was increased to 0.44 g/g, in the case of Ilmen et al. [18], and to 0.57 g/g in the case of our strain. While fermenting xylose without neutralizing agent, our strain yielded 0.58 g/g, and theirs yielded 0.3 g/g. This demonstrated that in the case of the strain constructed in this study, medium neutralization is not essential to obtain good product yields, but it is beneficial, and it prevents lactate being used as a carbon source after glucose or xylose are depleted. Furthermore, we demonstrate that raising the incubation temperature to 32 °C increases lactate yield by 30% (to 0.64 g/g on glucose without medium neutralization). This underlines the need to consider, as a priority, the optimum temperature of heterologous enzymes which originate from bacteria and not necessarily the growth optimum for the yeast host.

It is noteworthy that both the wildtype and the Lac^+^-engineered strain exhibit similar growth curves despite the suboptimal pH conditions of the medium. Typically, the optimal pH range for the growth of *S. stipitis* is between 4.5 and 5.5 [32]. However, during L-lactate production by the Lac^+^ strain, the pH of the medium without the addition of calcium carbonate tends to fall below 4 (Figure 2, Figure 4 and Figure 6). Generally, the medium was about 2 pH units more acidified during growth of the Lac^+^ producer than during growth of the wildtype. The pH value of the medium is the lowest at the same point at which the lactate concentration in the medium is the highest. The ability of the Lac^+^ strain not only to survive but also to continue producing L-lactate under such acidic conditions underscores the potential utility of this strain in industrial applications in which controlling and maintaining strict pH conditions can be challenging. This adaptability could reduce the need for frequent pH adjustments during fermentation, thus enhancing the cost-effectiveness of the process. The growth curves of both strains under the same conditions are fairly similar, especially on glucose, suggesting that the metabolic engineering aimed at L-lactate production has been achieved without compromising the fundamental cellular growth characteristics. This is a crucial consideration, as maintaining robust growth while introducing new metabolic pathways is essential for the commercial success of genetically engineered strains.

Notably, both our study and that of Ilmén et al. [18] show that lactate-producing *S. stipitis* does not produce ethanol as a side product during xylose fermentation, unlike the wildtype strain. However, during glucose fermentation, both the wildtype and lactate-producing strains produce ethanol as a side product (Figure 5). Since this phenomenon was observed in two independently constructed strains expressing L-lactate dehydrogenases from different bacterial sources and under distinct regulatory elements, it is clear that this effect is not strain-specific. Ilmén et al. [18] hypothesized that ethanol is produced but rapidly consumed as a carbon source, rendering it undetectable. However, we provide experimental evidence disproving this hypothesis by showing that ethanol is not quickly used as a carbon source during xylose fermentation. Ethanol was added to the medium at the beginning of fermentation, and its concentration remained constant until the last time point (Figure 5).

**Figure 8:**
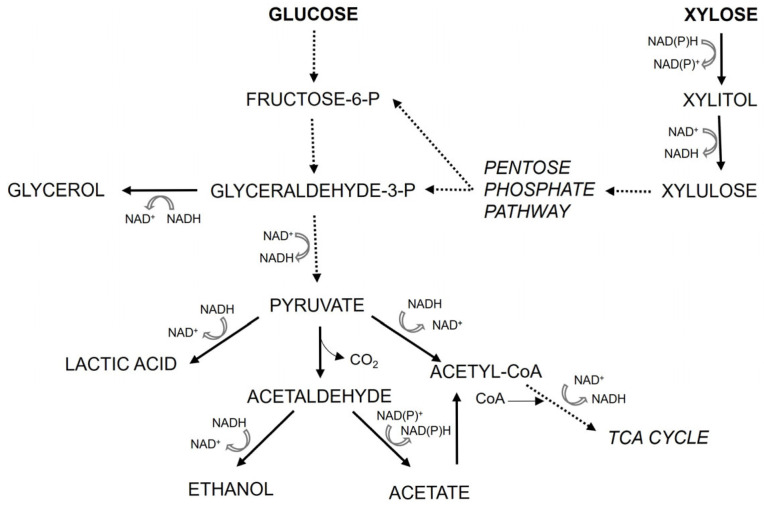
Schematic representation of central carbon metabolism in *S. stipitis* engineered for lactate production. In this work, concentrations of glucose, xylose, glycerol, lactate, ethanol, and acetate were measured.

Although we cannot yet fully explain why ethanol is absent during xylose fermentation in these lactate-producing strains, the data suggest that genes directly or indirectly involved in ethanol production are regulated differently depending on the carbon source (xylose vs. glucose) and possibly dependent on the presence of lactate (as suggested by Turner et al. [33], who noticed a similar effect in *S. cerevisiae* engineered to use xylose and produce lactate). In a broad sense, this could be any gene involved in glucose and xylose uptake and metabolism, pyruvate decarboxylase, alcohol dehydrogenase, and enzymes in the TCA cycle. Figure 8 shows a schematic representation of a part of the central carbon metabolism of a yeast capable of utilizing both glucose and xylose, as well as producing ethanol and lactate, as is the case in the Lac^+^ strain engineered in this study. Feng and Zhao [34] compared the glucose and xylose metabolism of wildtype *S. stipitis* and *Saccharomyces cerevisiae* engineered for xylose utilization (*S. cerevisiae* does not naturally ferment xylose). In general, they concluded that on xylose in *S. stipitis*, less carbon fluxes are diverted towards the TCA cycle than in *S. cerevisiae* and that on xylose, *S. stipitis* exhibits a respiro-fermentative metabolism (both respiration and fermentation with ethanol production occur). They stressed that *S. stipitis* had a superior fermentation effect on xylose compared to engineered *S. cerevisie*, which outlines the importance of using this yeast as a platform for bioethanol and biochemical production on renewable lignocellulosic hydrolysates. On glucose in *S. stipitis*, the direction of carbon flux towards the TCA cycle is more pronounced than in *S. cerevisiae*, which is expected, since *S. stipitis* is a Crabtree-negative yeast, and *S. cerevisiae* is Crabtree-positive (consistent with [35]). Nonetheless, the *S. stipitits* control wildtype strain still produces ethanol on xylose (both our strain, as well as that of Ilmen et al. [18]). The data of Feng and Zhao [34] also show that there are differences in NADH requirement and utilization when *S. stipitis* is growing on xylose or glucose. Lactate and ethanol synthesis both use NADH, i.e., both reactions influence the redox balance in the cell in the same manner. However, lactate is produced directly from pyruvate, while production of ethanol from pyruvate is a two-step reaction, and NADH is required at the second step. This might steer pyruvate towards lactate production if NADH is limited. Overall, to definitively conclude what the reasons behind the phenomenon noticed in this and earlier work [18] are, further investigation of carbon fluxes and redox balance in lactate-producing *S. stipitis* is needed. Regardless of the underlying mechanism, from a biotechnological point of view, the lack of ethanol as a side product during lactate production process is a huge advantage of such strains.

## 5. Conclusions

Overall, the natural ability of *S. stipitis* to grow and ferment pentoses (which is not a feature of *S. cerevisiae* yeast) and even co-ferment xylose and glucose, to some extent, combined with genetic modifications enabling lactate production, make the yeast strain constructed in this work an interesting resource for further development of sustainable L-lactate production.

## Data Availability

The original contributions presented in this study are included in the article/Supplementary Materials. Further inquiries can be directed to the corresponding authors.

## Supplementary Materials

The following supporting information can be downloaded at https://www.mdpi.com/article/10.3390/jof11060413/s1, Figure S1: Sequences of optimized Idh1 and Idh2 genes with native *S. stipitis* promotor–terminator pairs used for expression; Figure S2: Southern blotting analysis of *S. stipitis* transformants.
