# Supplementary material for: Production of Lactate by Metabolically Engineered Scheffersomyces stipitis
Source: J Fungi (Basel). 2025 May 27;11(6):413. doi: 10.3390/jof11060413 (PMC12194211; doi:10.3390/jof11060413)

SUPPLEMENTARY FIGURES

Supplementary figure S1. Sequences of optimized *IdhL1* and *IdhL2* genes with native *S. stipitis* promotor-terminator pairs used for expression. The *ldhL1* (green) and *ldhL2* (blue) genes, optimized for expression in *Candida* yeasts, were fused to the regulatory regions of the *TDH3* (red) and *PDC1* (orange) genes, respectively. Black sequences are restriction sites added to facilitate fragment cloning.

5'GGCCGCACTCACGGTGTTCAGCTCATGTGAATCGGTTTTAGATTCGGCCGACGTTGTGGCCGATATGAACGAAATTGGTTCAT
TCTGTAGCTTGCGAAAAATAGAAGTTACTCAGGATACTTCGACGCCCCGTTTCTGAACGCAAAGCCAAAAGGGGGTCTGTTCT
GTGGGAATTTTTCACTTCCCCCCAGAAGTACAATTGTGAGATCATAAGATACGCTCCTTATGACATCAACCACCTAAGCCAAGA
ACTGTTGCGAAACCAATTGTTGACGTTGGTGGTCTTATTCAATGCTGCAGACGTACATGATGCATGGCTAGCGGCTTCTACAAG
AATAAGGCGATTCCATGAATACATCATCATCTGTGAAATGGCTTCGGGCTCTTCTCGAGATGGGTTCTTAGAGGCTCGGTGTTAG
CCGCCCAATCGAGTAATATCTGCGACACACCAACGCTAACTTGACCGACGGAATAAAACCGACGGTATAAGACCGAGCCACGG
CTGCAAGTGGATGGCCATTTTGAATCCAAGATGATGTAACTCCGCATCGCAAGAAGTCCACAATTGAATGGCGAGAACTACGA
CATCTGTAGAAGCCCTCATGACGACGTGAATAGGAGGCGAGAAGGTTCTAGAAACGCTTTCGTGTGTGGAACAAGCGGTAATG
GTGTGACATTGCAAAAAGAGGATCCAAGAAAGAGAGCGCAAATCGGCCGTTGAAGATGAAATCAAAAAAGGGGCTCTTTTCT
GGGCAAAGAGCTCGAATCCTGGTAGTGAAATTGAATTGCCTAATTTTCGTGGTGTAGGCCCAGCCAAAGTTGAGTGGTTGCGA
AAAATACCAAACCGAATTCAACAAACCGAATTGTAAATTCGGACTTTGTTGCTGAAATGCCGGCCTGGGTTTAAGGGCTTCTCT
GAATGGGGCAGGCTTATTGCTCCAAGCTTGTAGCACGGTACCATAGAGCCCGGTGACAGCCGTTTGGGCCCGGAATTGCCAGC
GGTCTACATGTTGCACCTGGCTCGGTTACAACCGGTTACTGTTCGAAGAGCAGGAACAGGCAACTAGGTTGGGGTAAAGCCCG
GAGTCGGTAGGATGGATACTGGCCAGGTTCTACCGCCGAGGTCCTCAGCGGACAAAAGGCACATGATCGGCCCAAGCTGTAAT
CCAGGCTCAAGCTGTAAACCAAGGGGCGCCAGGTAGCGAGTAATCACGTGGTCTGAGACTGGATGGTACAGACAGGACTTAG
AGGATTAGACGGCCAGAGAAGTAAACCGGACTTGGCGTGGATTAAAGCTGGTGGAAATTTGGAGGGTTCTGCTGTCGGCGAG
AGGATGCTATGACGTCGGCAGGCAGAGGCCGCCACATGGAGACCAGCCAAATGAGTAATTCGGACTCTGATGGCAGGCTGGC
ATATAAGCCTCGGTGTCGGAGTTCTGTAATGCGTTGGAGCGAGGATTTGCAGGTTGGAGTATTGTGTGATGAGATGAAGTTGCA
ATGGGAGACCGTGTCAAGAGCATGTAGATAAGAGCAAGTAGAGAAGAGCAGGTAGAGCAGAGTGACTCGAGCAGAGTAGAC
TGGAGCCGTAGGATTACTCAGTTCTGGAGGAAATGTGACAACCAAAGTGACCAAAAATTGAGGTATTAATGGCTGAAAAATTT
GAGATGACTCTGTAGAAAGTTGAGTCAAATGCTGATTAATTTGGTTCTATTATGCCTCTCGTAGAAGATTGCAAAAGAGCAACT
GGATGAGGTGCTATCAAGTGATGCGAAGAGAACCTGCAAACAGGCCAGAGTACATGCCGTGGGTTGATCTCTGGTCGAGTGTG
CTGGCTACAGCCTTAAGTACGGAGAGTACAGCTACAGGGTGGTTTTTGCTGGGCTACAGCTACAGCATTGCAGTTTGAAGGTT
AGAGTGTAGAATGTAGCAGACGGCTTAAGGCTGGTGGAGTTTAGTCGAAACTCGTTAGTATTTCCGTGAAGGCAGCCATTGTG
AAAATTGAACATCACCTGAGGTATTTTAGCCACCAGAAGCGGCGGTACGGAAGAAAGTGTGTACAATGGTTGGTGGTGGAATT
GCGTGCATGCCTGATGGGGCAATATTAATTAGATAGAGCTTTGGTGATATTAGTGGATAATAGAATTCACAGAGAAGACATCAGG
AGCAATTTCCAAGAGCCATTGATGATGTAATTGCCCCAACAGCAAGATTCAGATCTGACAATTGACCACCGTTTTGTAGAAGCA
AAAAATCGTAGATTATCACCAAGAGGGTTTTTCACCGAACCAGCAAATAGAAACTATTCCGTAGAACTCGCCCAGGCTTTTTTG
CTAGCACTTTCCAGCAGTAGAACCGTCCAATTAAGTCAACAGGAACCATTGAGGTCGAGCCCAACCACCTGAACCCCCTCACG
GTCGTGTCCCTATTATTGATCCAGAGGGTGCCAGTTTCGGTAGCCAATATTGGTTCATGGGTTTCTATGGCCCGGAGTGAGTTTG
CAGGTTGGCCCCGGTGCCGTCTGCAGGATGGGAGTTATAGCGGCCAAACTTCACATTTCGAAATCCTGCTGCAGCCAATCTGA
AGAATTAATATAAATTCGTGTCGAATCGCCGTCTGTGAAATTTCAGTACTTGATTTTCTTTTCTTCTTCTTTTTCTCTTTTGTTTCT
TCAGAATCAATTCACATTTTTTCTTCCCTATAAACAATTCATCATGTCTGAGGAGAAAATCCACAAGATCATTTTGGTAGGAGAC
GGGGCCGTAGGTAGTACCTACGCCTTTTCATTAGTTCAGCAGGGTATCGCACAGGAACTTGGGATAGTAGATATAGTCAAAGAA
AGAACACAAGGGGACGCTATTGACTTGGCTGATGCCACCCCTTGGATAGCCCCAAAGACCATATATAGTGCAGAGTATTCCGAT
GCCAAGGACGCTGACTTGGTGGTCATTTCTGCAGGAGCCCCTCAAAAACCTGGAGAAACTAGATTGGACTTGGTAAATAAGA
ATCTTAAAATTTTATCTAGTATCGTAGAACCAATTGTCGAATCCGGTTTTAACGGTATCTTTCTTGTTGCCGCTAATCCTGTTGATA
TCTTAACTCACGCCACTTGGCGAATGTCAGGATTTCCTAAAGATCGAGTGATAGGTTCAGGAACTAGTCTTGATACAGGTCGAT
TGCAAAAGGTCATAGGAGAAATGGAGCACGTCGACCCACGTTCTGTAAACGCCTATATGTTAGGTGAGCACGGAGATACCGAG
TTCCCTGTTTGGTCATACAATAATGTTGGGGGAGTTAAAGTCTCCGACTGGGTTAAGGCCCATCCTGAGGTGGGAGAGAACAA
ATTAGAGGCTATACACAAGGAGGTTGCAGACATGGCTTATGATATAATCAATAAAAAAGGAGCTACTTTTTATGGTATCGGAACC
GCCCTTGCCTTTATAACCAAGGCTATCTTAAACAACGAGCACAGAGTGTTACCTCTTTCCGTTCCTATGGATGGTGAATATGGGC
TTCACGATTTGCATATCGGAACCCCTGCAGTAGTGGGTCGTCATGGTTTGGAGCAGGTGATCGAGATGCCATTAAGTGCCGACG
AGCAGGCTAAAATGGAAGCATCAGCAAAGCAGTTAAAAGAGGTCATGGACAAAGCATTCAAGGAGACCGGAGTGAAGGTAC
GACAATAACTATCCACGAAGTTGTAGGTCCACTGTGTGAACCTGGAGCTTCCGTGTGGTGATTAATTACCTATATATTCATACATA
TGAATTCATGAAAATGAGAAATATGATTAGTTGTAGATCGTAGAGAGAAGAATTACGAAGTACCGATTTCTGTAATGGAAGAGT
TTTCCAACGAAGAAGTTCTAGTTCGGTTTATTGACAAATAAGTTCTTTTATTCTTGTCTGACCCGATGCTCAGCTACTTTACCTTT
TCTACTCTTTCTACTCTACACTGTCCTTTCTACTTCTCTCAGTTCCTATTCCTGTTCTTCCTTTTGTCTCGCTCTCATCTTATCTGTG
ACGCACCTCATCTCATCAGAGTTAGCCACATATGACACAATTGACACAATTGGCCTGATCAGAGCCCGAAACCATCATAAAAAG
CAAAGTCCCTCTCGACCGAACTCGCTGACCAAAAATGGGGAGTCAATGGCTTTGTTTGGCTCATCTACATGAATTACTAATAGG
TGGATACCCCTAGTCATTTAAAAAACGTGTCGCAGTTGTGTCGCAAAAAAAGTTCGCCCGCGGTTTACAGACAGAGAAGCTAT
TTTAATGCGTAATGAATAATTAATGTTGACTTTTTGCAACCATTCAATACAATACAATGTAGTAGTCGTAGTAATCGGGAAAGATC
ATCTTCAAAAGCCAAGAAATGTCTGCCAAAACTGTCATGTCGTTTGAGCTAATACTCAAAGCAGGCACCTACTCTTTATTTTTTC
TCACATGAATAAAGTCGTCCAGTCTACAGTCAACTCTAAGTCATTGCTGGTTTTGCTGGAACATAATTTGAGTCCAGTCTAGATA
GCTACTCAATTTCGGAACAACTGGGTAAGCATGCAGGCTCACAGCGCTTTGCTTCCACTGCTTGCATTGTACAAAGGAGGCTC
CTCGAGGAAGAACTCTACGAACTGTGTAGAAGTGGGCGCATGGGAGCTGGCGTGAATTTCAATCATGGGTACGAAGTCATTGT
GACACTAGAGGCATTAGAAGTGCTGGAAGTAGGCATGAGAATTGGATGATGGGAGGACTTCTAAGAGCATGGTATTACTAGTAT
GCGTTTGACGGATTTCGTATTAATGGCTAGGGTTATTGCAAGTCCTCCGCGTGACATGCTCCATGCCTGCAACCAGCCTTTCAAG
ATTGAAAGCGAATGATGGATTCAATGTGGAAAATGGTCGTAATTAGAGTTGTGTGTTGCAGCAGGCGGGAAAATAATGAGCAG

AGCTAAAGCTGCAGGACGCAGATGCTTTGTAAGTGGATAATTGGTTTTTGTGTACTATGCGTTTAGACCAGCGGCGGAATGTAA
GGGTGGAATGACTCTGCTACGATTGTAGTAGTGACCCAGGCAACCATGCTAGAGAAGGGATCTCGGTGGTGAAACTAGATTAG
GGAGAATAGAGATCTACCATTTGGCAAGAACACTGCACAATTTCTACAATATCTAATGATGTAGCAGAAAGGGTTGCGATTGCG
GCTAGTGCTGAGTCTACTGATACCGCTACAGCTACCTGGCCATTCTGCGACCAGCGGGCGAAGTTGAAAGAGGCATACGGCAT
TTGTGGGGCACTTGTGGGAAATATAAGGTATCGGAGCGGTAATGCTGAAAAATAGTTGAAATATCGAATTAGGCACGGCTTAAG
TTTTTGCTTCGGCTTATGCGACTACTAAAGGTAAACAATAACCAAAAATTAAGGCCCAATTGCCTCGAGTATCGTGCTCCAGGA
TATGTGAGTTGGATTTCTCGACTACCCATTGACTATACCTGGTGGATCAGGCTTATTCGTTAAGTTAATCGCTCAATCAACTGATT
TTCCAGATCATGCGCTTAGCCAGGATGGAAAAAGTGCCAAAACGCGGAACTCACCCTGTGCGTCCGTGTGCAGGCTGCATTGC
AGGAGTTAGACTGGCCTACATCGGATGATATCCTCCCTCATTGCTTCAATAGGGGAGAGATGAGGTCTGGTGGATAATATTATTA
GTGGGGGATGCCGAATTCGAATTCAGGGATAAAGTATATAAAGGCCATTGGATCCTGGGAGGTTCGTCCCAGACATTTGGTTTT
GTTCTCCATCACAATCCAGATTTGATCAATTGACTACAATTAACAGAGTCTAGCCCTCCCGTATCCGGAAAAGCCTCTTCTCTGA
GATCGACTCAATTGACTTGAATAATATTTCAAAATATAAGAGCTTACTGCGAATCCCCCCATCCCCCAAAAAAGCCGCCTAACAT
ACAACTATCATGTCAAGAAGAAAAGTATTCCTTGTCGGGGATGGGAGAGTGGGATCAACATTTGCCAATGACTTACTTCAAAA
CGTCAAGATCGACGAATTAGTGATTTGCGATGTAGTTAAAAAAATTACTGAGGGAGACGCATTGGATTTAGAGGACCTTGCCCC
ATTCGTTGGGCAATGTACTGTAAAATCCGGTGATTACTCCGACGCAAAAGACGCTGATATAGCCGTGATTACCGCTGGGGCAGC
TCGTAAACCTGGAATGACAAGATTGGATTTAGTAAATACAAACGTGAAGATACTTGAGAGTATCGTAAAGCCTATCGTTGATAG
TGGGTTCAATGGAATCTTCGTAGTCTCTGCAAACCCAGTCGACATCTTGACTACCTTAACCCAAAAGTTGTCAGGTTTCCCAAA
AAATAAGGTCATTGGTACAGGGACATCCTTGGACACCGCTCGATTACGAGTTGCTTTATCACATAAAACAGGTGTTAACGTGGA
TCATATTGATGCCTATGTCTTAGGAGAGCACGGTGACACTTCTTTCGAGAATTTTGACGAAGCAATAATCGACCACAAACCATT
AAGATCTTATAAGGAATTGGATGAGCAAACATTAGCAGAGTTGGAGACCGACGTTCGAAAGAAGGGGGGGAAGATCATCGCC
AACAAAGGTGCTACATTTTACGGAGTGGCAATGTGCCTTACTCAGATATGCAAAGCTATCCTTGAGAATACTGCAAGAGTGATG
CCATTGTCTGCCCCAATGACTGGAGAGTATGGGATTCACGATTTGTATTTAGGTTCACCTGCCGTGGTTACAGCCAATGGTATTT
CAGATGTCATTGAGTTACATCTTTCAGAGGACGAAAAAAAGAAGATGACTTACTCAGCCACTAAAATGAAAGAAGTAGTAGAT
GGAATCAATTTATAATAAAAAGGATTAGTGTTATTTTTTTGCACCCACGTTATATATATTAATGCACAACTATGAGAGACAATTCC
GTAGTAAACGAGGACGCTTTTCTTTCAGATAAACTGCATTTTGTCTATCCGACAGATTTCGTATTTCATACGGCTTTGGCATAAAT
GCTCGATATGATTATTCTAGTAAATATTAGTAGGATCAGAGTGATCCACGGTAAAGGGGTTGCAAGGCTTACTAAATACGGAGAA
GCCGCACAATGGAGATACGGTCATCGTACCTCCGGAACTCCACCGCGCGTCGGAACACTGTGCCAACATCCATGACAAATTCC
GGATATTTAATCGGTGAACTTTTCACAAATTCATAAATTAATTTCAGGCTTTTTCATTTATCTTCTTTCCTTCTTTTGTCTTTACTTA
CAAGATCGATTGAAATCACAAACATACACTCACACCCGGG3'

Supplementary figure S2. Southern blotting analysis of *S. stipitis* transformants. Wells 1, 2, 3 and 4 contain DNA that has been digested with the restriction endonuclease PshAI. S1 – standard (NEB 1 kb DNA standard); S2 – standard (DNA of bacteriophage λ digested with HindIII); 1 – DNA used for transformation; 2, 3, 4 – DNA from *Lac*$^{+}$ transformants. DNA complementary to the *coHyg*^{R} region was used as a hybridization probe.

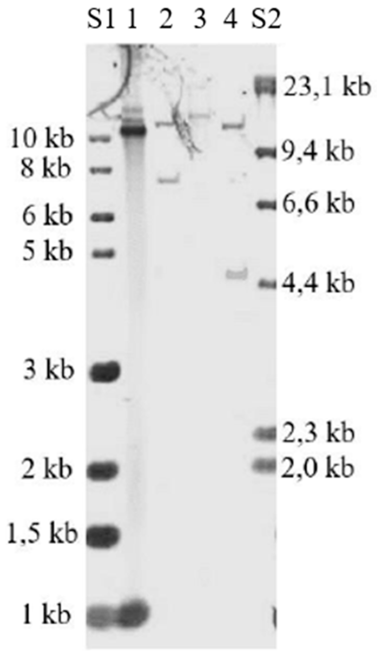

Supplement: Supplementary file 1 [file jof-11-00413-s001.zip › jof-3621111-supplementary.pdf]
